# Urolithin A attenuates memory impairment and neuroinflammation in APP/PS1 mice

**DOI:** 10.1186/s12974-019-1450-3

**Published:** 2019-03-14

**Authors:** Zhuo Gong, Jingyi Huang, Biao Xu, Zhenri Ou, Le Zhang, Xiaohong Lin, Xiujuan Ye, Xuejian Kong, Dahong Long, Xiangdong Sun, Xiaosong He, Liping Xu, Qingqing Li, Aiguo Xuan

**Affiliations:** 1 Institute of Neuroscience and Department of Neurology of the Second Affiliated Hospital of Guangzhou Medical University, Key Laboratory of Neurogenetics and Channelopathies of Guangdong Province and the Ministry of Education of China, Guangzhou, 510260 China; 2 Department of Neurology of the Sixth Affiliated Hospital, Guangzhou Medical University, Guangzhou, 511518 China

**Keywords:** Alzheimer’s disease, Urolithin A, Neuroinflammation, Memory impairment, Neurogenesis

## Abstract

**Background:**

Alzheimer’s disease (AD) is a progressive neurodegenerative disorder characterized by an abnormal accumulation of amyloid-β (Aβ) plaques, neuroinflammation, and impaired neurogenesis. Urolithin A (UA), a gut-microbial metabolite of ellagic acid, has been reported to exert anti-inflammatory effects in the brain. However, it is unknown whether UA exerts its properties of anti-inflammation and neuronal protection in the APPswe/PS1ΔE9 (APP/PS1) mouse model of AD.

**Methods:**

Morris water maze was used to detect the cognitive function. Terminal deoxynucleotidyl transferase-mediated dUTP nick end labeling (TUNEL) assay was performed to detect neuronal apoptosis. Immunohistochemistry analyzed the response of glia, Aβ deposition, and neurogenesis. The expression of inflammatory mediators were measured by enzyme-linked immunosorbent assay (ELISA) and quantitative real-time polymerase chain reaction (qRT-PCR). The modulating effects of UA on cell signaling pathways were assayed by Western blotting.

**Results:**

We demonstrated that UA ameliorated cognitive impairment, prevented neuronal apoptosis, and enhanced neurogenesis in APP/PS1 mice. Furthermore, UA attenuated Aβ deposition and peri-plaque microgliosis and astrocytosis in the cortex and hippocampus. We also found that UA affected critical cell signaling pathways, specifically by enhancing cerebral AMPK activation, decreasing the activation of P65NF-κB and P38MAPK, and suppressing Bace1 and APP degradation.

**Conclusions:**

Our results indicated that UA imparted cognitive protection by protecting neurons from death and triggering neurogenesis via anti-inflammatory signaling in APP/PS1 mice, suggesting that UA might be a promising therapeutic drug to treat AD.

## Introduction

Alzheimer’s disease (AD) is a multifaceted neurodegenerative disorder that causes cognitive deterioration and has no effective cure. The histopathological hallmarks of AD are the increase in neuronal amyloid-β (Aβ) plaque formation, hyperphosphorylated tau protein, neuroinflammation, and neuronal loss [1]. Aβ accumulation has been shown to recruit activated glia [2]. Indeed, reactive gliosis is increasingly regarded as an important player in the neuropathological processes of AD. Activated microglia and astrocytes have been reported to produce a wide range of proinflammatory factors such as IL-1β and IL-6, which increased amyloid precursor protein (APP) expression and Aβ deposition in models of AD [3, 4]. Conversely, activated glia also promoted the phagocytosis of the Aβ oligomer [5]. These findings show that activated glia have dual and opposing roles with respect to neuroinflammation in Aβ pathology. Numerous epidemiological reports supported the finding that anti-inflammatory therapy can reduce the risk for AD by more than 50% [6, 7]. Thus, anti-inflammatory therapy has been proposed as a potential therapeutic strategy for AD.

Ellagic acid (EA) is a hydrolyzed form of ellagitannins (ETs), which are abundant in pomegranate, berries, and nuts. Complex dietary EA and ETs are poorly absorbed in humans, but they get further metabolized by gut microflora to yield a series of urolithins [8]. Among the urolithin species, urolithin A (UA) is the major metabolite observed in humans [9]. Several studies have demonstrated that UA had anti-inflammatory and antioxidant properties in vitro and in vivo [10–14]. However, it is unknown whether UA improves cognitive function and attenuates neuroinflammation in the APPswe/PS1ΔE9 (APP/PS1) transgenic mouse model of AD. Therefore, the aim of this study was to determine if UA can rescue cognitive impairment in APP/PS1 mice and to elucidate the underlying cellular and molecular mechanisms of its effects.

## Methods and materials

### Animals and drug treatment

Female APP/PS1 transgenic mice were purchased from The Jackson Laboratory (Strain name: B6C3-Tg (APPswe, PS1dE9) 85Dbo/J; No. 004462). Age- and gender-matched wild-type (WT) littermates were used as controls. The mice were allowed to adapt to the laboratory environment before testing. The experiments were carried out in compliance with The Guidelines for Animal Care and Use of China, and the experimental protocols were approved by the animal ethics committee of Guangzhou Medical University.

Mice (28 weeks old) were orally administered 300 mg/kg UA (Standard, China) dissolved in 0.5% carboxymethylcellulose at the same time each day for 14 days. Control mice (APP/PS1 transgenic mice and wild-type mice) were orally administered the same quantity of 0.5% carboxymethylcellulose (vehicle).

### Morris water maze

After UA treatment, the spatial learning and memory of mice were assessed by the Morris water maze. Briefly, the maze consisted of a stainless steel pool (120 cm in diameter and 50 cm in height) with a submerged escape-platform (10 cm in diameter) placed 1 cm below the water surface. The water temperature was maintained at 24 ± 1 °C. The spatial learning task consisted of four consecutive days of testing with four trials per day. In each trial, the time required to find the hidden platform was recorded as the escape latency. The mice were given a maximum of 60 s to find the hidden platform. If a mouse failed to locate the platform within 60 s, the session was terminated, a maximum escape-latency score of 60 s was assigned, and the mouse was manually guided to the hidden platform (10 s). To test spatial memory, a single probe trial was conducted 24 h after the last trial of the fourth day. The submerged platform was removed and the mice were placed into the pool from the quadrant opposite to the quadrant where the platform used to be (target quadrant). The mice were allowed to freely swim for 60 s. The time spent in the target quadrant and numbers of crossings through this quadrant were recorded. Swimming speed was also recorded. All of the behavioral parameters of the mice were tracked, recorded, and analyzed using SMART 3.0 software (Harvard Apparatus).

### Immunohistochemistry and immunofluorescence

After the behavioral tests, the mice were anesthetized and transcardially perfused with phosphate-buffered saline (PBS; Boster, China). The brains were removed and post-fixed with paraformaldehyde overnight. They were then incubated in 30% sucrose in PBS for cryoprotection, and 30-μm serial sections were cut using a cryostat. Next, the sections were incubated with 0.3% H_2_O_2_ in methanol for 10 min, followed by a blocking solution of 10% normal goat serum in PBS for 20 min. For immunohistochemistry, the sections were incubated with primary antibody (anti-NeuN (1:200, 24307, CST, USA), anti-Aβ40 (1:500, 44047, NOVUS, USA), anti-Aβ42 (1:200, 14974, CST, USA), and anti-Iba-1 (1:500, 100-1028, NOVUS, USA) at 4 °C overnight and then 37 °C for 30 min. After being washed in PBS the following day, the sections were incubated with biotinylated anti-mouse or anti-rabbit secondary antibodies (Boster, China) in PBS for 30 min at 37 °C. They were then incubated with avidin-biotin peroxidase solution (SABC kit, Boster, China) and colorized with a 3,3′-diaminobenzidine (DAB) kit (Boster, China). For immunofluorescence, the sections were incubated with anti-DCX (Doublecortin, 1:200, 4604, CST, USA), anti-Aβ (anti-Aβ, 1:200, 8243, CST, USA), and anti-GFAP (Abcam, 1:800, 4674, Abcam, USA) in the blocking solution at 4 °C overnight. The following day, the sections were washed three times in PBS and incubated with Alexa 488- or Alexa 594-conjugated IgG secondary antibodies (Invitrogen, CA, USA) at room temperature for 2 h. Nuclei were counterstained by incubation in 1 μg/ml 4′,6-diamidino-2-phenylindole (DAPI) (Solarbio, China) for 15 min followed by exhaustive washing in distilled water. Coverslips were mounted in Gel Mount (VECTASHIELD, CA, USA), and the sections were inspected under a scanning confocal microscope (Leica, Germany).

Quantitative analysis of immuno-positive cells present in the sections was carried out under microscopic magnifications (Olympus, Japan) and was assessed from six random fields of view in each section (three sections per animal) using CellF software (Olympus). In each animal, one coronal section was taken from the anterior (− 1.22 mm from bregma), one from the middle (− 1.70 mm from bregma), and one from the posterior hippocampus (− 2.80 mm from bregma). The data are presented as the mean number of positive cells/mm^2^ in the tiled images. For the quantification of GFAP-positive cells, cell identity was ascertained by DAPI localization. Double-labeled cells positive for GFAP and DAPI were counted per mm^2^ from six random fields of view in each section under a fluorescence microscope (Olympus, Japan). The Aβ staining area (%) was calculated relative to the total area of the analyzed region (% area = plaque area/total area selected × 100). All of the cell counting was performed in a blinded fashion.

### Terminal deoxynucleotidyl transferase dUTP nick end labeling detection

The in situ terminal deoxynucleotidyl transferase dUTP nick end labeling (TUNEL) technique was performed according to the manufacturer’s instructions for the apoptosis kit (Roche, Basel, Switzerland). Briefly, sections were immersed in 0.3% H_2_O_2_ and then incubated for 120 min at 37 °C with TUNEL-labeling buffer, followed by 30 min at 37 °C in the avidin-biotin peroxidase solution. Next, the sections were rinsed in PBS and incubated for 10 min with DAB substrate solution. Three coronal sections were used for analysis, where the number of TUNEL-positive cells was manually counted in the cortex and hippocampus of the ipsilateral hemisphere. Imaging and cell counting were conducted using an Olympus light microscope (Olympus, Japan), and the resulting data are presented as the number of TUNEL-positive cells/mm^2^.

### 5-Bromo-2′-deoxyuridine labeling

5-Bromo-2′-deoxyuridine (BrdU) (Sigma-Aldrich) was administered intraperitoneally as a single injection of 100 mg/kg per day for 1 week preceding the behavioral tests, and a remainder of 100 mg/kg 24 h immediately before the animals were killed. For BrdU labeling, the non-specific binding sites were blocked by incubation in a blocking serum (bovine serum albumin 3%, Triton X-100 0.3%) for 10 min and then incubated with anti-BrdU (Abcam) at 4 °C overnight. The following day, brain sections were incubated for 1 h at room temperature with secondary antibody (FITC; Abcam) dissolved in the blocking serum. Nuclei were counterstained with DAPI. Coverslips were mounted in Gel Mount (Vectashield, CA), and the sections were inspected under a scanning confocal microscope (Leica, Germany).

### ELISA

Brain tissue was homogenized in RIPA buffer, sonicated briefly, and centrifuged. The supernatants were collected and quantified for soluble Aβ40 (DAB140B), Aβ42 (DAB142), IL-1β (DY401–05), IL-6 (DY406–05), and TNF-α (DY410–05) using enzyme-linked immunosorbent assay (ELISA) kits (R&D Systems, USA) according to the manufacturer’s instructions.

### Real-time reverse transcription-PCR

Total RNA from hippocampal and cortical tissue was extracted using TriZol reagent (Invitrogen, USA). Reverse transcription was performed with an ExScript RT Reagent Kit (Takara Bio Inc., China). Real-time PCR analysis was conducted using SYBR Premix Ex Taq (Takara Bio Inc., China). The PCR primer sequences were as follows: IL-1β (Accession number: NM_008361): 5′-AATGCCACCTTTTGACAGTGAT-3′ (sense) and 5′-TGCTGCGGGATTTGAAGCTG-3′ (antisense); IL-6 (Accession number: NM_031168): 5′-AGGATACCACTCCCAACAGACC-3′ (sense) and 5′-AAGTGCATCATCGTTCATACA-3′ (antisense); TNF-α (Accession number: NM_013693): 5′-CACGTCGTAGCAAACCACC-3′ (sense) and 5′-TGAGATCCATGCCGTTGGC-3′ (antisense); β-actin (Accession number: NM_007393): 5′-GCTGTGCTATGTTGCTCTAG-3′ (sense) and 5′-CGCTCGTTGCCAATAGTG-3′ (antisense).

The PCR parameters included an initial denaturation at 95 °C for 30 s, followed by 39 cycles of 95 °C for 5 s and 60 °C for 1 min. The relative gene expression was normalized to the mean expression level of β-actin.

### Western blotting

The expression levels of p-AMPK, p-P65NF-κB, p-P38MAPK, Bace1, and APP were analyzed by Western blotting. The protein samples were heated at 100 °C for 5 min with a loading buffer containing 0.125 M Tris-HCl (pH 6.8), 20% glycerol, 4% SDS, 10% β-mercaptoethanol, and 0.002% bromophenol blue. The samples were then separated by sodium dodecyl sulfate-polyacrylamide gel electrophoresis (SDS-PAGE) and transferred onto polyvinylidene (PVDF) membranes. The membranes were incubated with 3% bovine serum albumin (BSA) in Tris-buffered saline with Tween (TBST) (10 mmol/L Tris at pH 7.5, 150 mmol/L NaCl, 0.05% Tween-20) and probed with corresponding primary antibodies (anti-p-AMPK, anti-p-P38MAPK, anti-p-P65NF-κB, anti-BACE1, and anti-APP; Cell Signaling Technology, Boston, USA) at 4 °C overnight. After incubation with horseradish peroxidase-coupled secondary antibodies for 2 h at room temperature, the protein bands were quantified by densitometry (Syngene, UK).

### Statistical analysis

All of the values are expressed as the mean ± standard error of the mean (SEM). For the Morris water maze experiments, the escape latency during the spatial learning tests was determined by a two-way repeated-measures analysis of variance (ANOVA) with Student-Newman-Keuls post-hoc tests. All of the other experiments were analyzed using one-way ANOVA followed by Bonferroni’s post-hoc test. *P* < 0.05 was considered significant.

## Results

### UA ameliorates learning and memory deficits in APP/PS1 mice

**Fig. 1:**
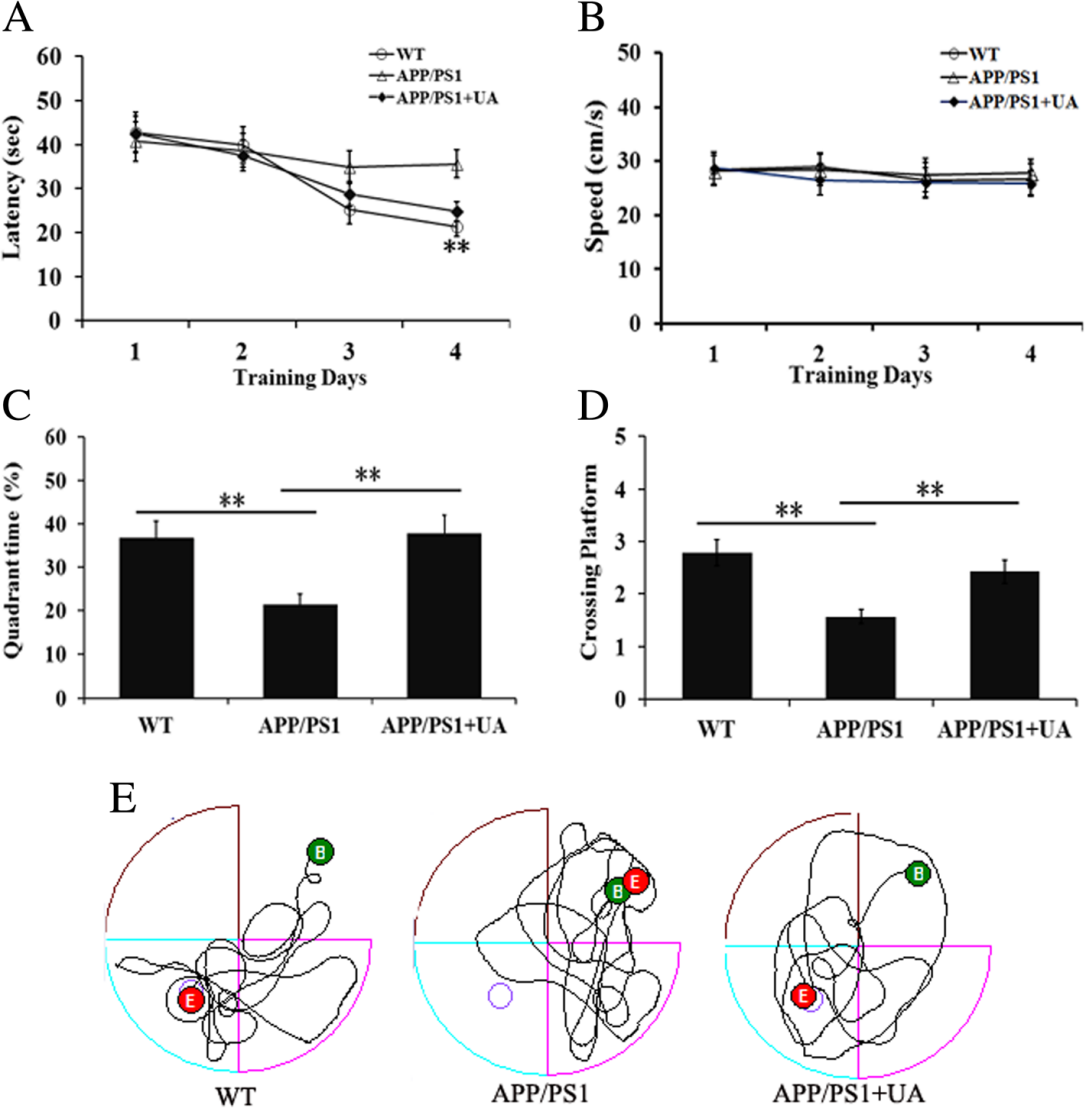
UA counteracted cognitive decline in APP/PS1mice. **a** The escape latency in the spatial learning test. **b** The average swimming speed among all the groups. **c** The percentage of time spent in the target quadrant in the probe test. **d** The number of crossings through the target quadrant (where the platform was previously located) in the probe test. **e** Representative path tracings in each quadrant during the probe trial. All data are presented as the mean ± SEM, *n* = 8–10 mice per group. **P < 0.01; *WT* wild-type, *UA* urolithin A

To investigate the potential therapeutic benefit of UA for the cognitive function of APP/PS1 mice, we conducted the Morris water maze to assess spatial learning and memory ability. We found that APP/PS1 mice spent more time locating the platform (escape latency) compared with wild-type (WT) mice, indicating a significant cognitive decline in terms of spatial learning. In addition, there was a significant difference in escape latency between UA-treated and vehicle-treated APP/PS1 mice, implying that cognitive function in terms of spatial memory was significantly improved by UA treatment (Fig. 1a). Swimming velocity remained stable among the three groups, suggesting that UA treatment did not influence the locomotor activity of mice (Fig. 1b). These findings showed that UA attenuated spatial learning deficits in APP/PS1 mice.

To test the effect of UA on spatial memory consolidation, probe trials were performed to assess the maintenance of spatial memory. Compared with WT mice, APP/PS1 mice took longer to reach the location of the missing platform and crossed the target quadrant less often (Fig. 1c–e). However, UA-treated APP/PS1 mice showed significantly more time spent in the target quadrant and increased crossovers compared with vehicle-treated APP/PS1 mice (Fig. 1c–e). These results indicated that UA ameliorated the spatial memory of APP/PS1 mice.

### UA prevents cell death in APP/PS1 mice

**Fig. 2:**
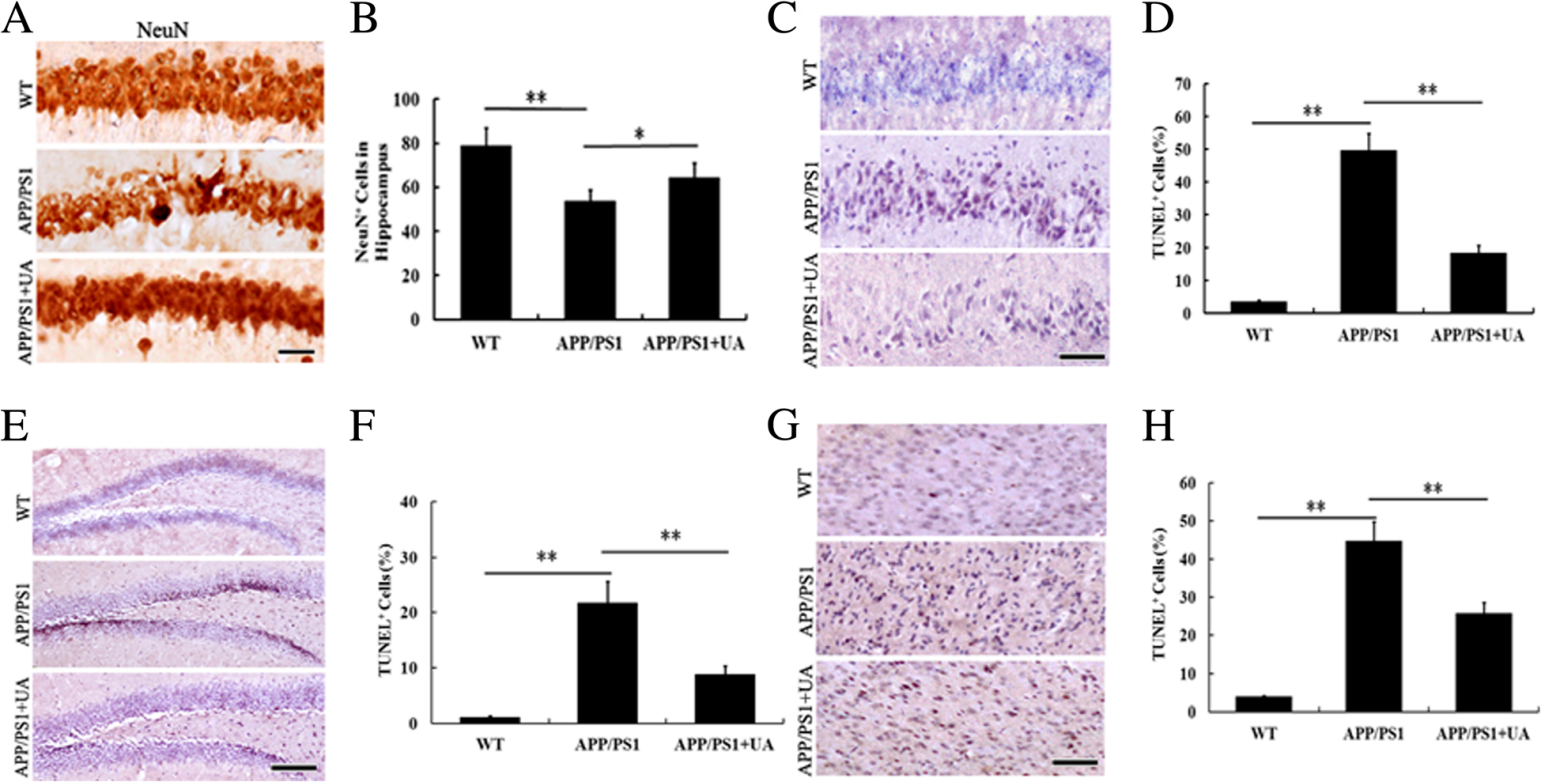
UA alleviated neuronal death and enhanced hippocampal neurogenesis in APP/PS1 mice. **a**, **b** Immunohistochemical analysis of NeuN^+^ cells in the CA1 regions of different treatment groups. Scale bar = 100 μm. *n* = 8–10 mice per group, three sections per animal. **c**–**f** Quantitative analysis of TUNEL-reactive cells in the CA1 and dentate gyrus (DG) regions of the hippocampus. Scale bar = 200 μm. *n* = 8–10 mice per group, three sections per animal. **g**, **h** Quantitative analysis of TUNEL-reactive cells in the frontal cortex. Scale bar = 200 μm. *n* = 8–10 mice per group, three sections per animal. Data are presented as the mean ± SEM. *P < 0.05 and **P < 0.01. *WT* wild-type, *UA* urolithin A

Neuronal degeneration and loss are regarded as the main contributors to the cognitive decline in AD patients [15] and APP/PS1 mice [16]. To assess whether UA can attenuate cell death, we measured the change in the number of NeuN-positive (NeuN^+^) cells in the hippocampus. Our data showed that UA treatment prevented the loss of NeuN^+^ immunoreactivity in the CA1 region of hippocampus of APP/PS1 mice (Fig. 2a, b). Results from the terminal deoxynucleotidyl transferase dUTP nick end labeling (TUNEL) assay further confirmed that UA significantly reduced cellular apoptosis in the cortex and hippocampal CA1 of APP/PS1 mice (Fig. 2c–h). These findings indicated that UA prevented cell death in the cortex and hippocampus of APP/PS1 mice.

### UA enhances hippocampal neurogenesis in APP/PS1 mice

**Fig. 3:**
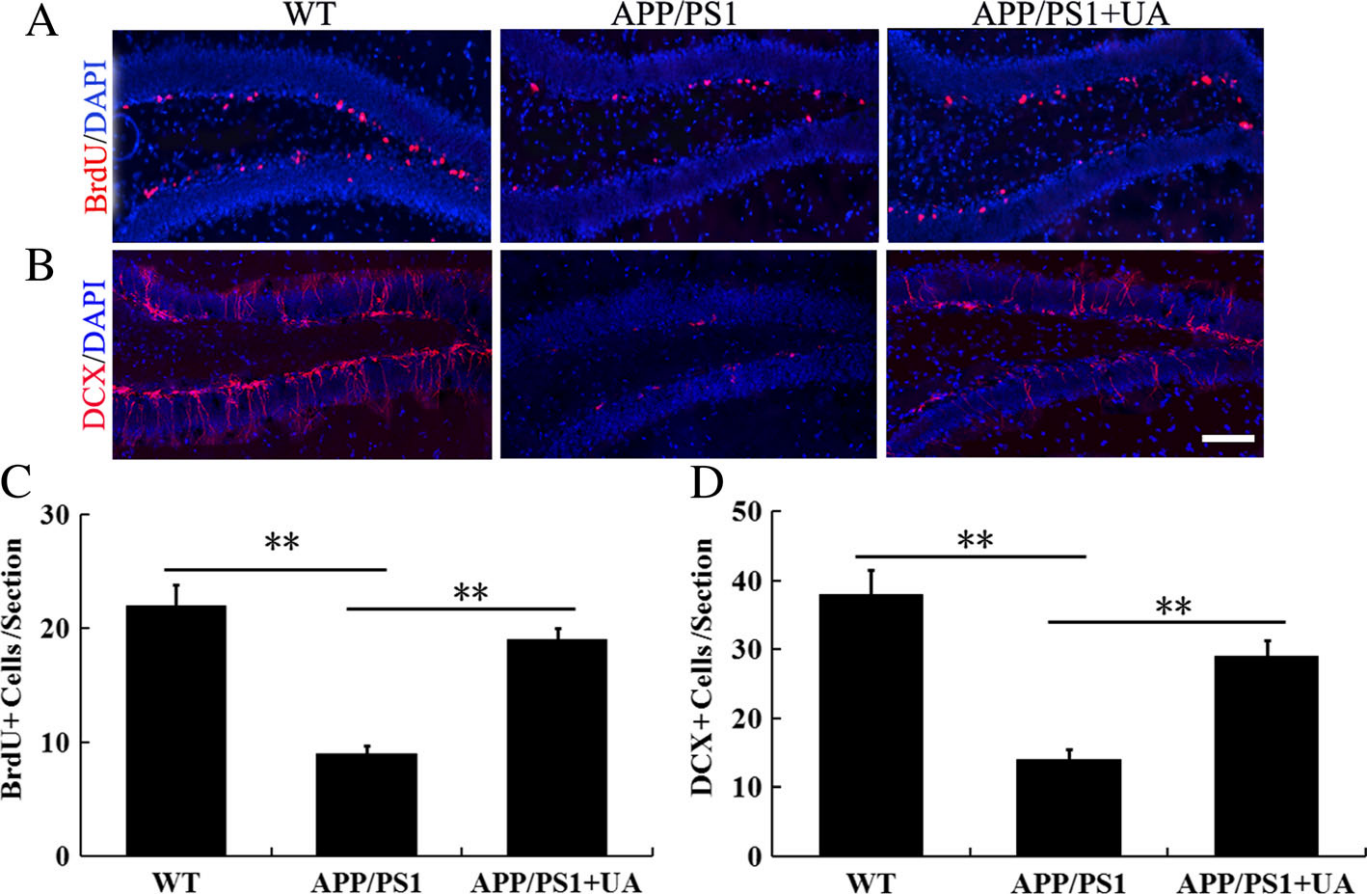
UA-enhanced hippocampal neurogenesis in APP/PS1 mice. **a** Representative fields of BrdU-positive cells in the hippocampal dentate gyrus. Scale bar = 200 μm. **b** Representative fields of DCX-positive cells in the hippocampal dentate gyrus. Scale bar = 200 μm. **c** BrdU-positive cells in each group. **d** Quantification of DCX-positive cells in each group. *n* = 8–10 mice per group, three sections per animal. Data are presented as the mean ± SEM, *P < 0.05 and **P < 0.01. *WT* wild-type, *UA* urolithin A

Hippocampal neurogenesis plays an important role in hippocampal-dependent learning and memory [17]. To determine whether UA increases hippocampal neurogenesis, we performed bromodeoxyuridine (BrdU) and doublecortin (DCX) staining. Our results showed that significantly more BrdU-positive (BrdU^+^) cells were seen in the UA-treated AD mice than in the vehicle-treated AD mice (Fig. 3a, c). In addition, compared with WT littermates, there were significantly fewer DCX-positive (DCX^+^) cells in the dentate gyri of vehicle-treated AD mice and significantly more in the dentate gyri of UA-treated AD mice (Fig. 3b, d). These results demonstrated that the diminished capacity for neurogenesis in APP/PS1 mice was at least partially restored by UA treatment.

### UA alleviates plaque burden and Aβ levels in APP/PS1 mice

**Fig. 4:**
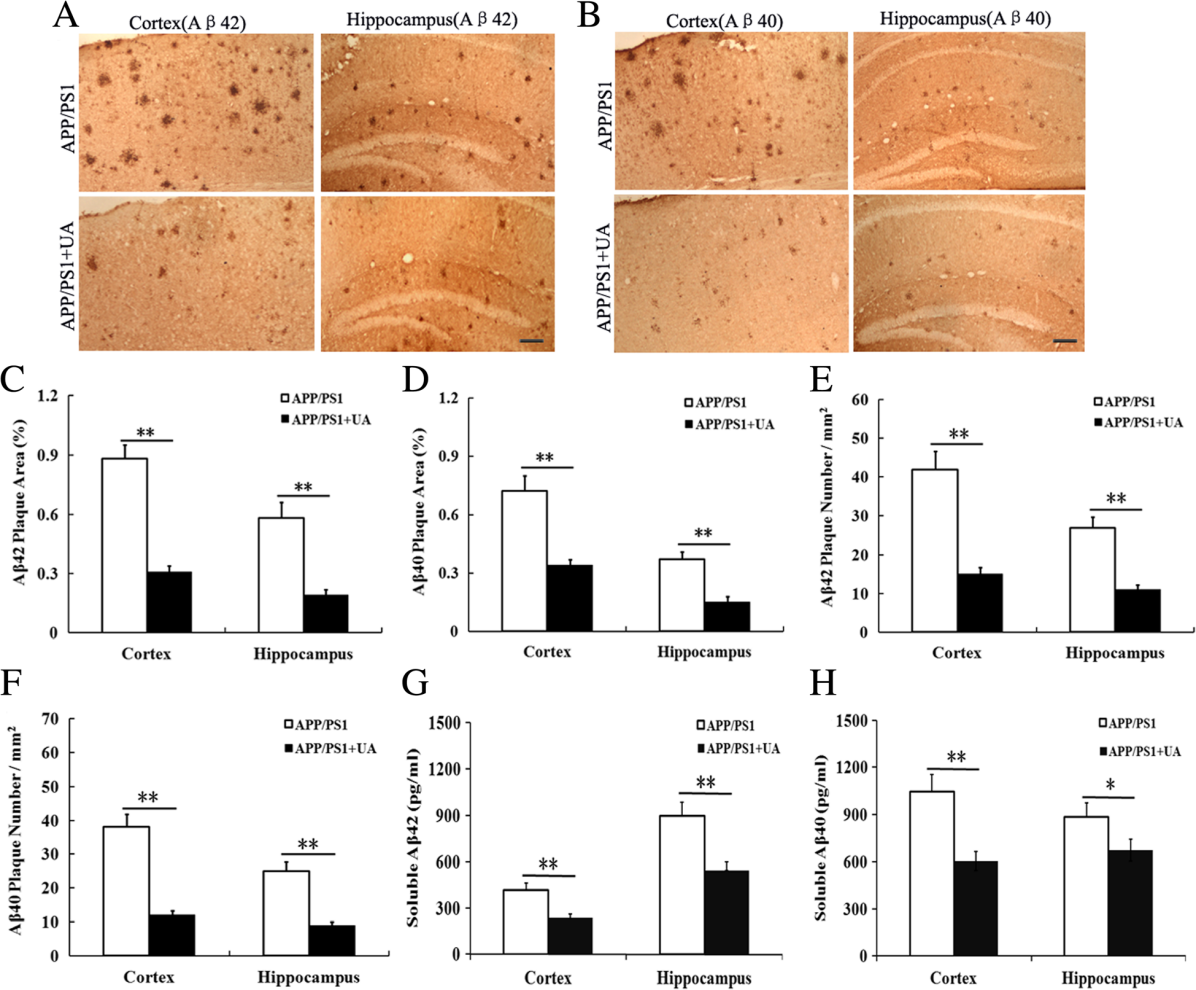
UA alleviated brain Aβ burden in APP/PS1 mice. **a** Representative images of Aβ42 deposits in the cerebral cortex and hippocampus. Scale bar = 250 μm. **b** Representative images of Aβ40 deposits in the cerebral cortex and hippocampus. Scale bar = 250 μm. **c**, **d** Quantification of Aβ area in the hippocampus and cerebral cortex, *n* = 8–10 mice per group, three sections per animal. **e**, **f** Quantification of the number of Aβ deposits in the cerebral cortex and hippocampus. **g**, **h** Levels of soluble Aβ40 and Aβ42 were measured by ELISA, *n* = 4 mice per group. Data are presented as the mean ± SEM, *P < 0.05 and **P < 0.01. *WT* wild-type, *UA* urolithin A

To explore the effect of UA on the levels of Aβ plaque deposits, immunohistochemistry with antibodies specific to Aβ40 and Aβ42 was performed on fixed brain tissue. The mean area covered by Aβ40-positive and Aβ42-positive plaques was markedly higher in the cortex and hippocampus of APP/PS1 mice compared with WT mice (Fig. 4a–d). However, UA treatment significantly decreased the mean area containing Aβ plaques in APP/PS1 mice (Fig. 4a–d). In addition, we also quantified the density of Aβ40- and Aβ42-positive plaques in the cortex and hippocampus. Our results demonstrated that UA significantly reduced the plaque number/mm^2^ of Aβ40-positive and Aβ42-positive plaques compared with APP/PS1 mice (Fig. 4a, b, e, f), suggesting an inhibitory effect of UA on Aβ deposition.

Next, we performed an enzyme-linked immunosorbent assay (ELISA) to quantify Aβ levels in the cortex and hippocampus of the AD mice. Our results showed that soluble Aβ40 and Aβ42 levels were high in APP/PS1 mice (Fig. 4g, h), but UA treatment significantly reduced the levels of soluble Aβ40 and Aβ42 in the cortex and hippocampus compared with the APP/PS1 group (Fig. 4g, h). These results demonstrated that UA decreased Aβ levels in APP/PS1 mice.

### UA attenuates reactive gliosis in APP/PS1 mice

**Fig. 5:**
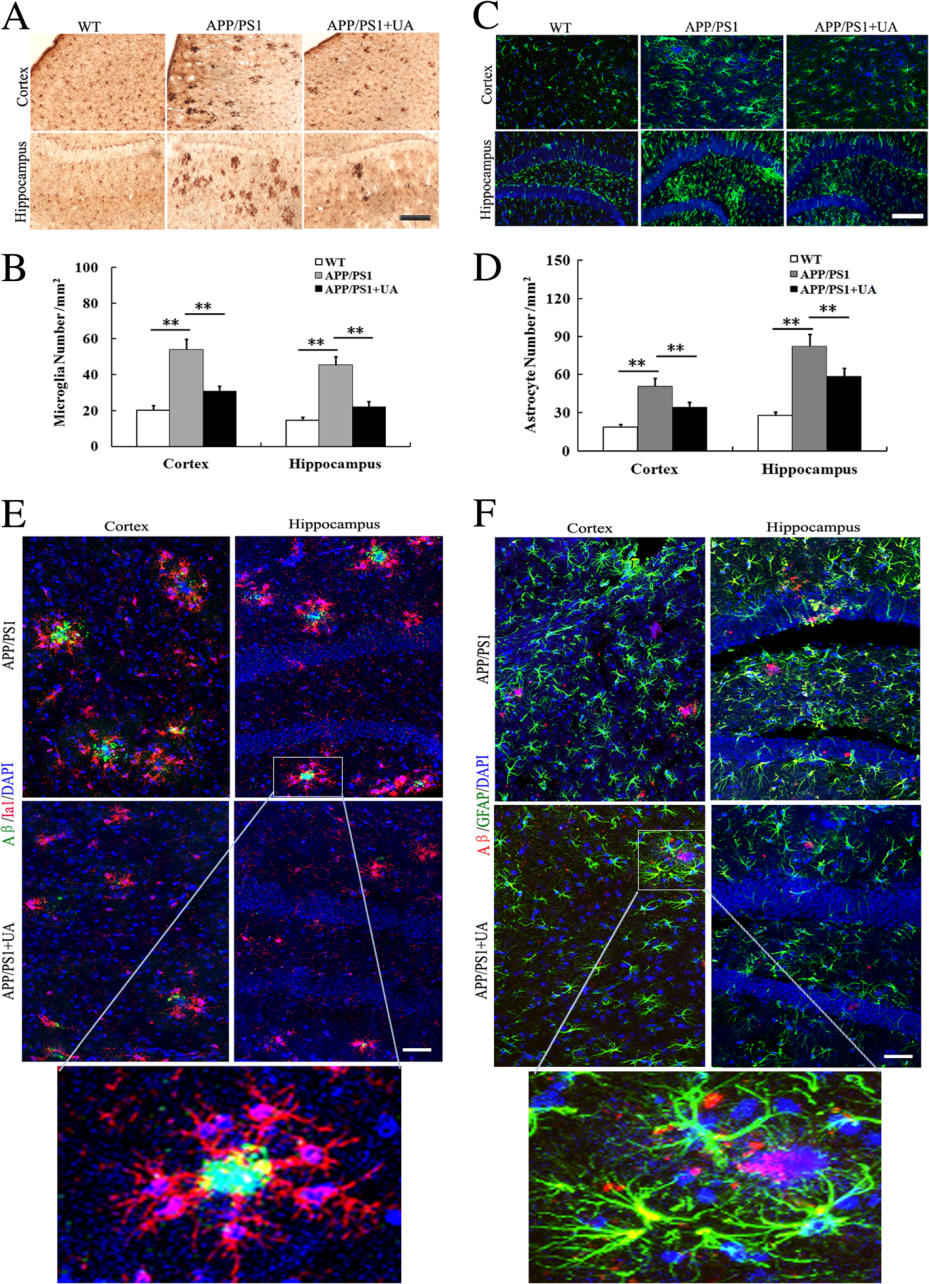
UA decreased glial reactivity in APP/PS1 mice. **a** Representative immunostaining of Iba-1 in the cerebral cortex and hippocampus of the different groups. Scale bar = 200 μm. **b** Quantification of microglia number in the cortex and hippocampus. **c** Colocalization of fluorescent GFAP (green) and DAPI (blue) in the hippocampus and cortex. Scale bar = 200 μm. **d** Quantification of astrocyte number in the cerebral cortex and hippocampus. **e** Colocalization of fluorescent Aβ (green), Iba1 (red), and DAPI (blue) in the cerebral cortex and hippocampus. Scale bar = 50 μm. **f** Colocalization of fluorescent Aβ (red), GFAP (green), and DAPI (blue) in the cerebral cortex and hippocampus. Scale bar = 50 μm. All of the quantified data are presented as the mean ± SEM, *n* = 8–10/group, three sections per animal, **P < 0.01. *WT* wild-type, *UA* urolithin A

A typical hallmark of the AD brain is the presence of activated astrocytes and microglia surrounding Aβ plaques, which contributes to the inflammatory process of brain injury [18]. To investigate the anti-inflammatory effects of UA on APP/PS1 mice, we assessed the microglial and astrocytic reactivity in the cortex and hippocampus by staining with antibodies against Iba1 and GFAP, respectively. The staining revealed that reactive astrogliosis and microgliosis were markedly observed in the cortex and hippocampus of APP/PS1 mice compared with WT controls. Moreover, staining for both activated microglia and astrocytes was heavy in the vehicle-treated APP/PS1 mice and significantly less intense in the UA-treated APP/PS1 mice (Fig. 5a–d). We also observed that Aβ plaques were surrounded by reactive microglia and astrocyte in the brains of vehicle-treated APP/PS1 mice, whereas reactive microglia and astrocytes were reduced around Aβ plaques in the brains of UA-treated APP/PS1 mice (Fig. 5e, f). These results indicated that UA effectively decreased microglia and astrocyte activation in APP/PS1 mice.

### UA decreases proinflammatory cytokine levels in APP/PS1 mice

**Fig. 6:**
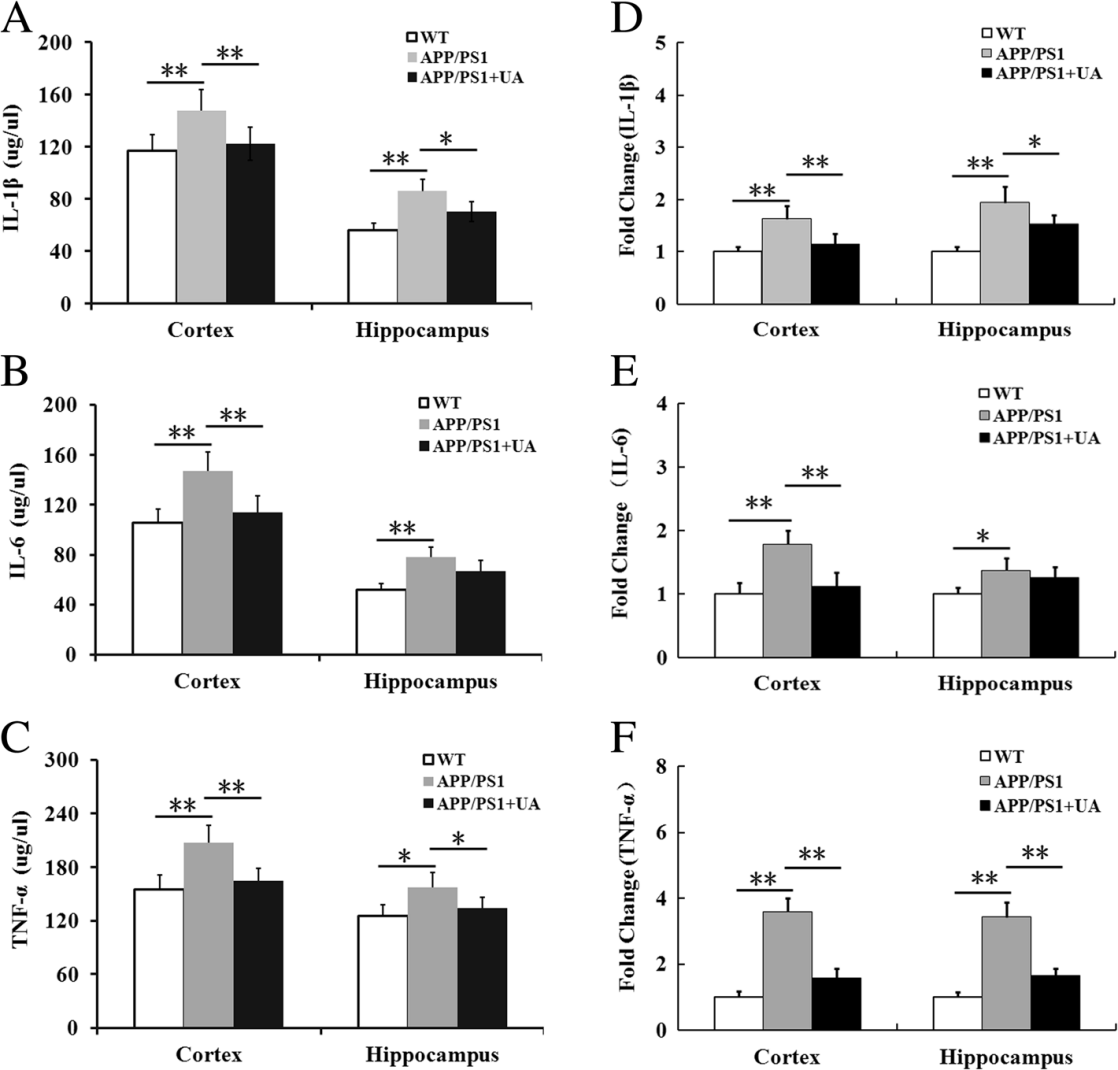
UA reduced proinflammatory cytokine production in APP/PS1 Mice. **a**–**c** IL-1β, IL-6, and TNF-α levels in the cortex and hippocampus were measured by ELISA. **d**–**f** The expressions of IL-1β, IL-6, and TNF-α mRNA in the cortex and hippocampus were detected by quantitative RT-PCR. Data are presented as the mean ± SEM, *n* = 4, *P < 0.05 and **P < 0.01. *WT* wild-type, *UA* urolithin A

Activated glia stimulated by Aβ has been reported to upregulate the expression of several proinflammatory chemokines and cytokines including IL-1β, IL-6, and TNF-α, which could result in an increase of Aβ production, neuronal death, and cognitive deficits [19]. Thus, we measured the expression of these three cytokines in the different experimental groups. Our results demonstrated that the levels of IL-1β, IL-6, and TNF-α were markedly increased in the cortex and hippocampus of the APP/PS1 group compared with the WT group (Fig. 6a–c). Interestingly, UA significantly reduced the levels of the inflammatory mediators IL-1β and TNF-α in both the cortex and hippocampus of APP/PS1 animals (Fig. 6a–c), suggesting that UA could inhibit the secretion of inflammatory cytokines. In addition, quantitative polymerase chain reaction (qPCR) results confirmed the above results (Fig. 6d–f). Taken together, these data indicated that UA effectively decreased the expression of proinflammatory factors released by activated glia.

### Multiple proteins participate in the working mechanism of UA in APP/PS1 mice

**Fig. 7:**
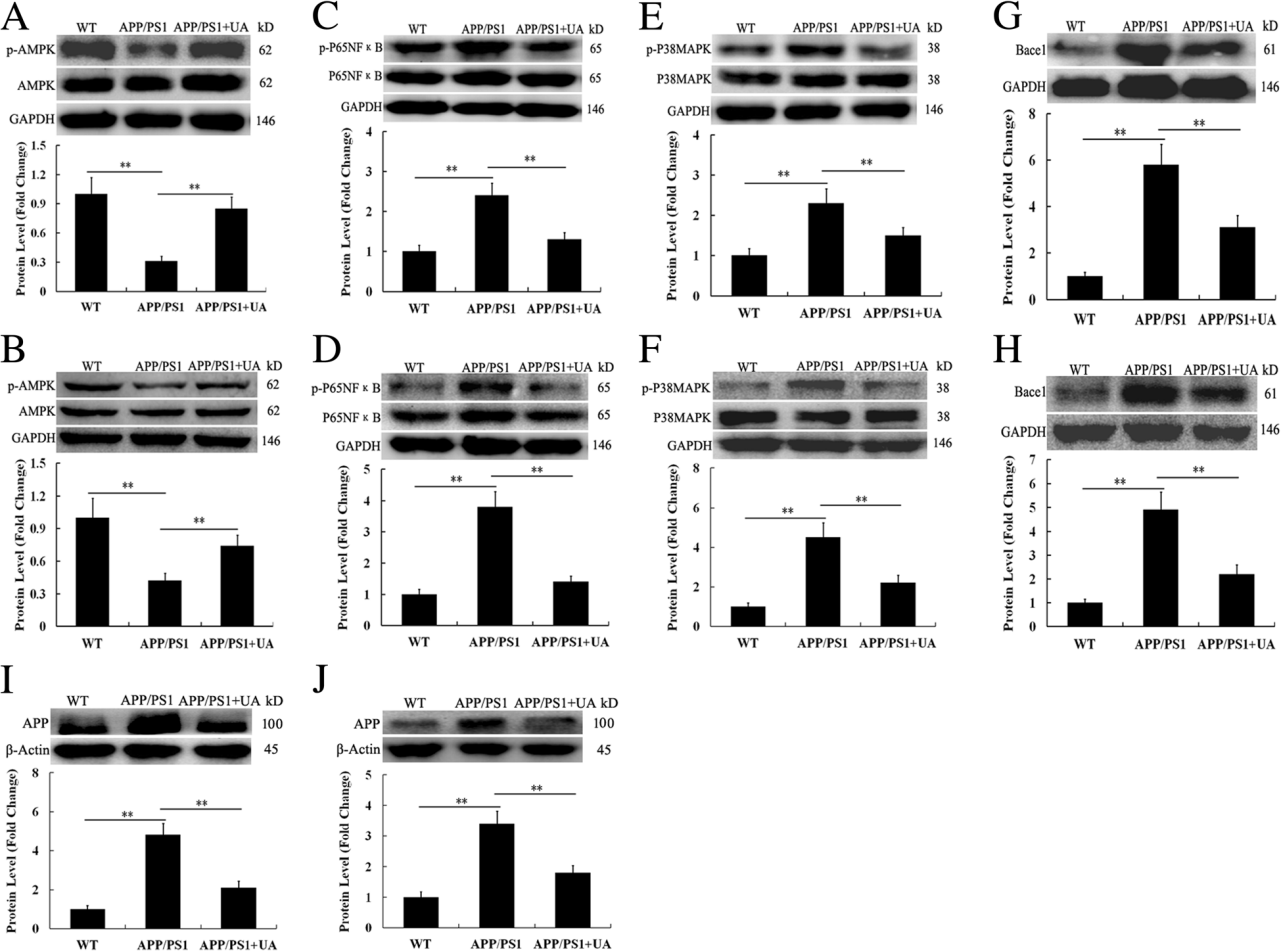
Effects of UA on the protein expression of p-AMPK, p-P65NFκB, p-P38MAPK, and Bace1, APP in the cortex and hippocampus of APP/PS1 mice. Western blot analysis of **a** p-AMPK in the cortex, **b** p-AMPK in the hippocampus, **c** p-P65NFκB in the cortex, **d** p-P65NFκB in the hippocampus, **e** p-P38MAPK in the cortex, **f** p-P38MAPK in the hippocampus, **g** Bace1 in the cortex, **h** Bace1 in the hippocampus, **i** APP in the cortex, and **j** APP in the hippocampus. For all the above Western blots, the relative optical density was normalized to GAPDH or β-Actin; *n* = 4; **P < 0.01. *WT* wild-type, *UA* urolithin A. The fold change refers to the ratio of p-AMPK/AMPK, p-P65NF-κB/P65NF-κB, p-P38MAPK/P38MAPK, BACE1/GAPDH, and APP/β-Actin compared to WT group

Previous studies have shown that 5′-AMP-activated protein kinase (AMPK)/Beta-site APP-cleaving enzyme 1 (Bace1) signaling was responsible for APP processing and Aβ production [20, 21]. It is well known that the release of proinflammatory factors requires nuclear factor kappa-light-chain-enhancer of activated B cells (NFκB) and/or the activation of the mitogen-activated protein kinase (MAPK) p38. AMPK/NFκB and AMPK/p38MAPK signaling pathways are involved in the decreased neuroinflammatory response [22, 23]. To further investigate the mechanisms involved in UA-mediated neuroprotection and anti-inflammation, the activation/phosphorylation of AMPK, p65NFκB, p38MAPK, and Bace1, APP proteins were studied. Our results showed that in the cortex and hippocampus of APP/PS1 mice, phosphorylated (p-) AMPK was markedly decreased compared with the WT group (Fig. 7a, b), whereas the levels of p-P65NFκB, p-P38MAPK, and Bace1, APP were notably enhanced compared with the WT group (Fig. 7c–j). Strikingly, after UA treatment, the APP/PS1 mice showed a remarkable increase in the expression of p-AMPK and a significant decrease in the expression of p-P65NFκB, p-P38MAPK, and Bace1, APP (Fig. 7a–j). Our findings indicated that the UA-induced alterations of these critical signaling proteins might contribute to its neuroprotective effect in AD.

## Discussion

AD is a neurodegenerative disorder characterized by progressive memory deficits and cognitive decline. Our findings in this study demonstrated that APP/PS1 transgenic mice exhibited severe memory loss. Recent reports have indicated that dietary supplementation with pomegranate extract (PE), which is ultimately metabolized by gut microflora to yield UA, ameliorated the loss of synaptic-structure proteins and improved behavioral performance in APPsw/Tg 2576 mice [24, 25] and APP/PS1 mice [26]. In contrast, another study reported that PE did not improve the cognitive performance of transgenic AD mice [27]. We speculated that the contradictory effects of PE may be attributed to its poor absorption, different active ingredients, and functional concentrations in vivo. In this study, APP/PS1 mice were intragastrically administered with UA. We found that UA treatment significantly ameliorated cognitive impairment. Our study also elucidated some mechanisms underlying the beneficial effects of UA in AD.

The brain in AD shows a selective and progressive degeneration of neurons, which can contribute to cognitive impairment [28]. Previous study has shown marked neuronal loss in both the CA1 field and dentate gyrus (DG) in APP/PS1 mice at 16 months of age [29]. No neuron loss was found in the neocortex of aged APPPS1 mice but a significant 11% neuron loss was found in the dentate gyrus of 17-month-old APPPS1 mice compared with age-matched control mice [30]. However, neuron loss is also reported for the hippocampal CA1 and frontal cortex but did not reach statistical significance in 12-month-old APP/PS1 mouse [31]. Amazingly, study shows that neuron death has been significantly enhanced in the cortex and hippocampus of 3-month-old male APP/PS1 mice compared with age-matched control mice [32]. In the current study, we found that 30-week-old APP/PS1 mice had substantial neuronal loss in the hippocampal CA1. Studies have confirmed that neuronal apoptosis is observed both in APP/PS1 mice [18] and AD patients [33]. Inhibiting hippocampal neuronal apoptosis relieved the cognitive dysfunction [34]. In our study, we found that UA significantly increased the number of NeuN-positive neurons and suppressed the apoptosis of hippocampal cells in APP/PS1 mice. These results indicated that UA not only repaired damaged neurons but also prevented hippocampal neuronal loss in APP/PS1 mice. Impaired hippocampal neurogenesis is involved in cognitive dysfunction. Some natural nuts and berries, when metabolized into UA, were shown to enhance mammalian hippocampal neurogenesis and improve cognition [35, 36]. Accordingly, we observed decreased neurogenesis in the hippocampus of APP/PS1 mice. Importantly, UA treatment of APP/PS1 mice significantly increased hippocampal neurogenesis, which might explain the improvement of learning and memory we observed in UA-treated transgenic animals.

Excessive Aβ aggregation into plaques is widely considered as one of the first changes that occur in the brain of AD [37]. Recent reports have shown that dietary supplementation with PE delayed the formation of senile plaques by decreasing the brain content of Aβ1–40 and Aβ1–42 [24, 26]. However, a different study showed that urolithins, but not PE or its predominant ellagitannins, prevented β-amyloid fibrillation in vitro [38]. In our study, we observed a marked increase of Aβ levels in the brains of APP/PS1 mice. Notably, UA treatment reduced Aβ deposits in the cortex and hippocampus of APP/PS1 mice, suggesting that UA inhibited the accumulation of Aβ deposits in APP/PS1 mice.

Previous studies have shown that neuroinflammation increased Aβ production, and that aggregated Aβ triggered microgliosis and astrogliosis, resulting in a proinflammatory state [39]. In our study, UA not only decreased the levels of activated microglia and astrocytes, but it also reduced Aβ levels in AD mice. It has been reported that urolithins possessed anti-inflammatory and antioxidative properties, with urolithin A exhibiting the strongest anti-inflammatory activity [10, 12, 40]. Therefore, we hypothesized that UA might suppress neuroinflammation and lead to the decrease of Aβ production. Accordingly, we found that UA treatment reduced the production of inflammatory cytokines in APP/PS1 mice, which suggested that UA may also affect glia. Previous evidence has demonstrated the infiltration of activated glia around Aβ plaques in AD brains [41], indicating that glia may provide the initial neuroprotective effect in AD pathology by phagocytosing Aβ. Consistent with the observations above, our results showed that UA might attenuate the Aβ burden in APP/PS1 mice by promoting glial phagocytosis (Fig. 5e, f).

The UA-mediated inhibition of neuroinflammation and neuronal apoptosis may contribute to the improvement of AD pathophysiology. However, the underlying molecular mechanisms are still largely unclear. Previous studies reported that PE activated AMPK in the hypothalamus [42], the liver, and adipose tissue [43]. Phosphorylated AMPK activated Nrf2, which promoted the expression of antioxidant proteins that protect against the oxidative damage triggered by inflammation [44]. In vitro data showed that UA attenuated triglyceride accumulation via AMPK activation in adipocytes as well as hepatocytes [45]. However, few studies have investigated whether AMPK is activated by urolithins in vivo. Our data addressed the question of the potential activation of AMPK by urolithins in vivo by showing that UA dramatically enhanced cortical and hippocampal AMPK activation in APP/PS1 mice.

Previous evidence also suggested that AMPK activation decreased Aβ production and could present a new potential therapeutic strategy in AD [46]. Activated AMPK has been reported to regulate the expression and trafficking of Bace1 in APP processing and Aβ generation [21]. Furthermore, AMPK activation increased autophagy signaling and facilitated lysosomal degradation of Aβ [47]. In our study, we found that the levels of Bace1 were reduced by UA treatment in APP/PS1 mice, indicating that AMPK/Bace1 signaling may be involved in the UA-induced decrease in Aβ deposition by reducing the cleavage of APP.

Activation and nuclear translocation of NFκB have been shown to elicit the release of proinflammatory cytokines, whereas inhibiting AMPK/NFκB signaling reduced the production of proinflammatory cytokines [22]. A large body of evidence has demonstrated that AMPK activation repressed NFκB signaling by activating SIRT1 [48], stimulating FOXO proteins [49], and suppressing ER stress [50]. Recent studies suggested that UA inhibited the phosphorylation and nuclear translocation of the NFκB p65 subunit, which reduced the expression of proinflammatory genes and diminished nitric oxide production [51, 52]. Consistent with this finding, we observed an obvious enhancement of NFκB phosphorylation in APP/PS1 mice, indicating that Aβ activated NFκB. More importantly, UA attenuated the levels of p-P65NFκB, which explains the anti-inflammatory effects of UA in APP/PS1 mice. In addition, P38MAPK has also been shown to regulate proinflammatory signaling networks and the biosynthesis of cytokines including TNF-α and IL-1β [53]. Studies have demonstrated that the activation of P38MAPK by Aβ occurred in the postmortem brains of AD patients and animal models, indicating that p38MAPK is involved in the pathogenesis of AD [54]. Inhibition of P38MAPK effectively alleviated the inflammatory response, Aβ deposits, and cognitive impairment in brains with AD [55, 56]. Thus, P38MAPK inhibitors are considered promising drug candidates for the treatment of AD. Whether the inflammation in AD is primarily induced through P38MAPK signaling remains unclear and requires further study. Many studies have provided evidence that UA decreased the phosphorylation levels of P38MAPK in LPS-stimulated microglia and IL-1β-treated human colonic fibroblasts [14]. However, another study revealed that UA increased the mRNA and protein expression of P38MAPK in HepG2 cells and bladder cancer cells [57, 58]. Our study found that activated P38MAPK was affected by UA treatment, with significant decreases in the levels of p-P38 observed in the brains of UA-treated APP/PS1 mice. Taken together, these findings suggest that suppression of P65NFκB and P38MAPK activity may contribute to the anti-inflammatory effect of UA.

## Conclusion

In summary, our results in an AD mouse model demonstrated the protective effects of UA on AD pathology by its targeting of multiple pathological processes such as reactive gliosis, inflammatory signaling, AB plaque formation, and apoptosis. Our findings indicate that UA may serve as a promising therapeutic agent for AD.

## Abbreviations

AD: Alzheimer’s disease
AMPK: 5′-AMP-activated protein kinase
Aβ: Amyloid-β
BrdU: 5-Bromo-2′-deoxyuridine
DAPI: 4′,6-Diamidino-2-phenylindole
DCX: Doublecortin
EA: Ellagic acid
ELISA: Enzyme-linked immunosorbent assay
ETs: Ellagitannins
MAPK: Mitogen-activated protein kinase
NeuN: Neuronal nuclei
NFκB: Nuclear factor-kappa B
PE: Pomegranate extract
TUNEL: Terminal deoxynucleotidyl transferase dUTP nick end labeling
UA: Urolithin A
WT: Wild-type
